# Cytocompatibility with osteogenic cells and enhanced *in vivo* anti-infection potential of quaternized chitosan-loaded titania nanotubes

**DOI:** 10.1038/boneres.2016.27

**Published:** 2016-09-20

**Authors:** Ying Yang, Haiyong Ao, Yugang Wang, Wentao Lin, Shengbing Yang, Shuhong Zhang, Zhifeng Yu, Tingting Tang

**Affiliations:** 1 Shanghai Key Laboratory of Orthopedic Implants, Department of Orthopedic Surgery, Shanghai Ninth People’s Hospital, Shanghai Jiao Tong University, School of Medicine, Shanghai, People’s Republic of China

## Abstract

Infection is one of the major causes of failure of orthopedic implants. Our previous study demonstrated that nanotube modification of the implant surface, together with nanotubes loaded with quaternized chitosan (hydroxypropyltrimethyl ammonium chloride chitosan, HACC), could effectively inhibit bacterial adherence and biofilm formation *in vitro.* Therefore, the aim of this study was to further investigate the *in vitro* cytocompatibility with osteogenic cells and the *in vivo* anti-infection activity of titanium implants with HACC-loaded nanotubes (NT-H). The titanium implant (Ti), nanotubes without polymer loading (NT), and nanotubes loaded with chitosan (NT-C) were fabricated and served as controls. Firstly, we evaluated the cytocompatibility of these specimens with human bone marrow-derived mesenchymal stem cells *in vitro*. The observation of cell attachment, proliferation, spreading, and viability *in vitro* showed that NT-H has improved osteogenic activity compared with Ti and NT-C. A prophylaxis rat model with implantation in the femoral medullary cavity and inoculation with methicillin-resistant *Staphylococcus aureus* was established and evaluated by radiographical, microbiological, and histopathological assessments. Our *in vivo* study demonstrated that NT-H coatings exhibited significant anti-infection capability compared with the Ti and NT-C groups. In conclusion, HACC-loaded nanotubes fabricated on a titanium substrate show good compatibility with osteogenic cells and enhanced anti-infection ability *in vivo*, providing a good foundation for clinical application to combat orthopedic implant-associated infections.

## Introduction

Titanium-based intramedullary nails have been widely used in the treatment of both closed and open tibial and femoral fractures; however, infections may occur, especially in the contaminated tibia and femur fractures.^1,2^ In a large series of tibial shaft fractures treated by intramedullary nailing, there was an incidence of postoperative infection of 1.9% for closed fractures and 7.7% for open fractures out of 1 106 cases.^3^ To remedy such an implant-associated infection, systemic administration of antibiotics and removal of intramedullary nails are the two conventional approaches, which may compromise patient compliance and lead to systemic toxicity, osteomyelitis, and bone nonunion.^4^ Thus, it is of great significance to endow the implants with anti-infection properties when the fracture fixation is at high risk of infection.

As an innovative modification of titanium implants, titania nanotube (TNT) arrays generated over a Ti surface by a simple and adjustable electrochemical anodization process^5^ have been extensively explored as a new antibiotic-loading method to mitigate the side-effects of systemic drug administration.^6,7^ Meanwhile, the nano-featured surface topography itself possesses improved antibacterial potential and osteoblastic activity.^8–11^ The most common microorganisms correlated with implant-associated infection are *Staphylococcus aureus* and *Staphylococcus epidermidis*, which can adhere to implant surfaces and form biofilms.^12^ Our previous *in vitro* and *in vivo* studies revealed that gentamicin-loaded nanotubes on titanium substrates exhibited evident antibacterial activity for the two bacterial strains mentioned above.^13,14^ However, the resistance to β-lactamase, aminoglycoside, lincosamide, trimethoprim, macrolide, tetracycline, and sulfonamide antibiotics in *S. aureus* and *S. epidermidis* is widespread in current orthopedics surgery.^15,16^ Therefore, implants with good antibacterial capability, especially under methicillin-resistant *stapthylococci* infection, is critical to the successful fixation and healing of bone fractures in current orthopedic surgery.

As a new water-soluble chitosan derivative, quaternized chitosan emerged to address the poor water solubility and restricted antibacterial capability of chitosan under an alkaline environment.^17–19^ As a cationic antimicrobial agent, quaternized chitosan has a broad spectrum of antibacterial activity caused by the electrostatic interaction between the positively charged quaternary ammonium groups of hydroxypropyltrimethyl ammonium chloride chitosan (HACC) and the negatively charged phosphoryl groups of the phospholipid components of bacteria membranes, which affected the cytoplasmic membrane integrity, eventually leading to cell death.^20,21^ In our previous reports, HACC-loaded polymethylmethacrylate exhibited obviously inhibited biofilm formation by antibiotic-resistant *staphylococci* both *in vitro* and *in vivo*.^22,23^ However, polymethylmethacrylate bone cement is not suited to intramedullary fixation of bone fractures. Thus, we further fabricated HACC-loaded nanotubes with various diameters on titania implants, which exhibited effectively inhibited bacterial adherence and biofilm formation *in vitro.*^24^

Although the *in vitro* antibacterial activity of HACC-loaded nanotubes with a diameter of 200 nm was reported to be better than those with a diameter of 160 nm,^24^ the cytocompatibility had a negative correlation with the diameter of the nanotubes.^13^ Therefore, we fabricated TNTs with 160 nm diameters on titanium plates and rods for the consideration of both the antibacterial capability and cytocompatibility in this study. Quaternized chitosan with a degree of substitution of 26% was loaded into these nanotubes to form an effective local antibiotic delivery system, and the *in vitro* cytocompatibility was assessed. Then, a methicillin-resistant *S aureus* strain was selected to establish an intramedullary infection model in rats and the *in vivo* anti-infection properties of the HACC-loaded TNTs coating were systematically evaluated. Meanwhile, titanium without modification (Ti), TNTs without drug loading (NT), and chitosan-loaded titania nanotubes (NT-C) were also evaluated and compared.

## Materials and methods

### Preparation of drug-loaded TNTs

TNTs with diameters of 160 nm were manufactured on Ti plates (10 mm in diameter and 2 mm thick) and rods (15 mm in length and 2 mm in diameter) at a constant voltage of 70 V for 1 h by electrochemical anodization treatment according to our previous reports.^13,24^ Chitosan (MW=50 000, 87% N-deacetylation) was purchased from Zhejiang Yuhuan Ocean Biochemistry Co., Ltd (Yuhuan, Zhejiang, China). Glycidyl trimethylammonium chloride (MW=151.63 g·mol^−1^) was purchased from Sigma-Aldrich (St. Louis, MO). Other chemicals used were of analytical grade. HACC with a quaternary ammonium degree of substitution of 26% and molecular weight of 5.0×10^4^ was prepared by combining chitosan and glycidyl trimethylammonium chloride, as reported in our previous work.^22^ Then, chitosan and HACC solutions of 20 mg·mL^−1^ dissolved in deionized water were loaded into the TNTs by lyophilization method and vacuum drying.^7^ In brief, 5 μL volume of drug solution was pipetted onto the surface of the nanotube, and then gently spread to ensure even coverage. The drug-loaded specimens were dried under vacuum in a freeze-drying system (Labconco 7753072; Labconco Corp, Kansas City, MO) at −45 °C for 2 h. The loading step was repeated for approximately 20 cycles until the nanotubes were loaded with 2 mg of chitosan and HACC according to our previous study.^24^ All prepared samples were sterilized with 25 kGy of ^60^Co irradiation before performing the *in vitro and in vivo* experiments.

### Characterization

Scanning electron microscopy (SEM, HITACHI SU8220, Tokyo, Japan) was used to verify the surface morphologies of the specimens. The surface wettability of the samples was determined by measuring the static water contact angles using the sessile drop method on a drop-shape analysis system (JC-2000D3, Shanghai Zhongcheng Digital Technology Co., Shanghai, China) at ambient temperature and humidity. Meanwhile, images were collected by the camera. Three measurements were performed at different points on each sample.

### Preparation of bacteria

Methicillin-resistant *S. aureus* (ATCC43300) was purchased in a freeze-dried form from the American Type Culture Collection (Manassas, VA). This strain was a biofilm-producing bacterial strain as verified by our previous study.^22^ Cells were suspended in phosphate-buffered saline (PBS) solution to 1×10^5^ CFUs·mL^−1^ after overnight trypticase soy broth culture. The minimal inhibitory concentrations of chitosan and 26% HACC against ATCC43300 were previously reported to be 64 μg·mL^−1^ and greater than 1 024 μg·mL^−1^, respectively.^22^

### *In vitro* cytocompatibility evaluation

Human mesenchymal stem cells, isolated and expanded as previously described,^25^ were selected to evaluate the cytocompatibility of these samples *in vitro*. A cell counting kit-8 (CCK-8) assay was used to analyze cell attachment at 4, 8, and 12 h. The cells were seeded into wells containing the specimens at a density of 4.0×10^4^ per cm^2^, and empty wells containing α-MEM and specimens served as blank controls. At each time point, a total of 50 μL of CCK-8 solution (Dojindo Molecular Technologies Inc., Kumamoto, Japan) was added to each well and then incubated for 3 h before reading at 450 nm, with 620 nm as the reference wavelength. The mean absorbance value (optical density) obtained from the blank control was subtracted from the optical densities of the tested groups. Meanwhile, the cells on the discs were also stained with 4,6-diamidino-2-phenylindole (Molecular Probe, Sigma Aldrich, St. Louis, MO, USA) for 15 min to observe the cell adhesion using a fluorescence microscope (Leica AF 6000, Heideberg, Germany).

Similar to the cell adhesion test, cell proliferation was also evaluated by the CCK-8 assay after 1, 4, and 7 days, and the seeding density of the cells was 2.0×10^4^ per cm^2^. The modified optical density values at 4 and 7 were normalized to those at day 1 in terms of the number of attached cells grown on different samples at day 1.^26^ The cell spreading and morphology on the discs were visualized by confocal laser scanning microscope (CLSM) (Leica TCS SP8, Leica Microsystems, Wetzlar, Germany), and the cell seeding procedures were similar to those of the cell attachment test. After 24 h of incubation, the samples were fixed with 4.0% paraformaldehyde for 15 min, and then the cells were permeabilized with 0.1% Triton X-100 in PBS for 10 min. After washing three times with PBS, the filamentous actin of the cell cytoskeleton was stained with rhodamine-phalloidin (Biotium, Hayward, CA) for 45 min, and then the nuclei were stained with 4,6-diamidino-2-phenylindole for 15 min.

In addition, the cell viability of various specimens was analyzed by a Live/Dead Cell kit (ab115347, Abcam, Cambridge, UK) as described previously.^27^ The cell seeding procedures were similar to those of the cell spreading assay. After a 24-h co-culture, cells attached on the specimens were stained with 500 μL of combination dye for 10 min, and were then detected by the CLSM (Leica TCS SP8). The viable cells with esterase activity appeared green, whereas dead cells with compromised plasma membranes appeared red, as described in the manufacturer’s protocol.

### Implant infection model in rats

The *in vivo* experimental procedures were approved and performed in accordance with the guidelines of the Animal Ethics Committee of Shanghai Ninth People’s Hospital. Forty-five specified pathogen-free grade 8-week-old male Sprague-Dawley rats (weighing 399.07±20.73 g) were randomly assigned to three independent groups (*n*=15 for each group). According to the previously reported surgical procedures,^14,28,29^ the left knee was exposed through a middle parapatellar incision after general anesthesia with an intraperitoneal injection of 1% pentobarbital sodium (100 mg·kg^−1^ body weight). The femoral medullary cavity was widened gradually with Kirschner rods to a diameter of 2.0 mm after the dislocation of the patellar. Then, 50 μL of PBS containing ATCC43300 at a concentration of 1×10^5^ CFUs·mL^−1^ was injected into the medullary cavity using a micropipette. Subsequently, a prepared Ti, NT-C, or NT-H rod was inserted. The opening cavity was sealed with bone wax, and then the surgical site was closed layer by layer. The rats were kept separate and allowed to eat and drink *ad libitum*. Fluorescence labeling was used to investigate new bone formation around the implants according to the established protocol.^30^ In brief, calcein green (20 mg·kg^−1^ body weight; Sigma-Aldrich) was injected subcutaneously at day 3 before sacrifice. The animals were killed after 42 days. No antibiotic was administered post-surgery.

### Clinical evaluation

All animals in the three groups were observed and recorded on the day of surgery and 3, 7, 14, 21, 28, 35, and 42 days after surgery. Body weight and temperature were selected as the observational indices and were obtained from all 15 animals in each group.^14,23^ Weight was measured on a precision scale (TCS, Shanghai, China), and body temperature was determined by a veterinary digital infrared thermometer (HRQ-S60, Zhengzhou, China). Knee joint swelling, skin exudation, and other inflammatory signs were regarded as the main local signs of infection.

### Radiographic evaluation

Lateral radiographs of the femur and knee joint were obtained at 3, 21, and 42 days after implantation. Radiographic manifestations were assessed on the basis of a modified scoring system.^23,31^ X-rays of the femurs and knee joints in all groups were read and interpreted in a blind manner by a radiologist unaware of the grouping characteristics and inoculum. Femurs were harvested and evaluated using a high-resolution micro-CT (SCANCO MEDICAL, μCT 80, CH-8306, Brüttisellen, Switzerland) at an isometric resolution of 20 μm (70 kV and 130 μA radiation source with 0.5 mm aluminum filter). Three-dimensional high-resolution reconstruction images obtained from the overall, longitudinal and transverse sections, the bone volume/total volume and the cortical bone mineral density of rat femurs were analyzed by the software (Image Processing Language, v4.29d, Scanco Medical AG, Brüttisellen, Switzerland) provided by the manufacturer.

### Microbiological assessment

Six rods in each group were aseptically explanted and rolled over trypticase soy agar for semi-quantification of bacteria adhesion on the rods on the day of sacrifice. The rods were placed in 5 mL PBS and then sonicated in an ultrasonic bath at 150 W (B3500S-MT, Shanghai, China) at a frequency of 50 Hz for 5 min, followed by rapid vortex mixing (Vortex Genie 2, Scientific Industries, New York, NY) at maximum power for 1 min to thoroughly dislodge the adhered bacteria.^14,29^ The adhered bacteria in the collected solutions after ultrasonication were measured by the spread plate method as previously reported.^13^ Then, the femurs after explantation were quickly frozen in liquid nitrogen and ground to a powder under sterile conditions.^23,29^ The bone powder was homogeneously vortexed in 5 mL PBS for 2 min. After centrifuging at 10 000 r·min^−1^ for 20 s, 50 μL of the supernatant was drawn and serially diluted 10-fold. The bacteria quantity in the bone powder was analyzed and expressed relative to the femur weight (CFUs·g^−1^ femur) using the spread plate method. CLSM and SEM were used for direct observation of biofilm formation on different rods after explantation. The rods were stained with 500 μL of combination dye (LIVE/DEAD BacLight Viability Kits, L7012; Thermo Fisher Scientific, Waltham, MA) and visualized by CLSM. Live bacteria with intact cell membranes appeared fluorescent green, whereas dead bacteria with damaged cell membranes appeared fluorescent red. Three-dimensional images were acquired from random rod positions. Meanwhile, the explanted rods were dehydrated by a series of graded ethanol solutions (30%, 50%, 70%, 80%, 90%, and 100%) for 10 min each. Subsequently, the rods were examined using SEM after critical-point drying and gold sputter-coating.

### Histopathological analysis

The remaining three femurs without implants of each group were fixed in 4% buffered formaldehyde for 2 days and decalcified for 1 week using Rapidly Decalcifier (DeCa DX-1000; Pro-Cure Medical Technology Co Ltd, Kwun Tong, Hong Kong) and then embedded in paraffin and cut using a microtome (CUT 6062; SLEE Medical, Mainz, Germany) to obtain 5-μm longitudinal and transverse sections. Hematoxylin and eosin and Masson’s trichrome staining were used to assess morphology, and Giemsa staining was used to assess bacterial contamination. Meanwhile, three undecalcified femurs containing rods were embedded in methyl methacrylate for infiltration and polymerization after fixation and graded dehydration. The embedded specimens were transversely cut at the middle and condyle of the femur (Leica SP1600 cutting equipment, Germany). The cut surface was glued to a slide, and sections were ground to a thickness of approximately 50 μm. The undecalcified sections were stained with Van Gieson to observe the morphology of the cortical bone and osteointegration around the implants. Slices labeled with fluorochrome were used to observe the new bone formation around the implants. Images were captured on a Leica DMI4000B (Leica Microsystems, Wetzlar, Germany).

### Statistical analysis

The data were expressed as the mean±standard deviations (mean ±SD). All *in vitro* experiments were repeated three times. One-way analysis of variance and the least significant difference test were used to evaluate the body weights, temperatures, and CFUs from the microbiological evaluations among the three independent groups. Nonparametric tests (the Mann-Whitney *U* test) were performed for comparison of the surface characteristics, *in vitro* cytocompatibility, and the radiographic evaluations among different groups. *P*<0.05 was defined as statistically significant, and *P*<0.01 was considered highly statistically significant. All statistical analyses of the data were performed using SPSS software (v19.0, IBM Corp, Armonk, NY).

## Results

### Surface characterization

**Figure 1:**
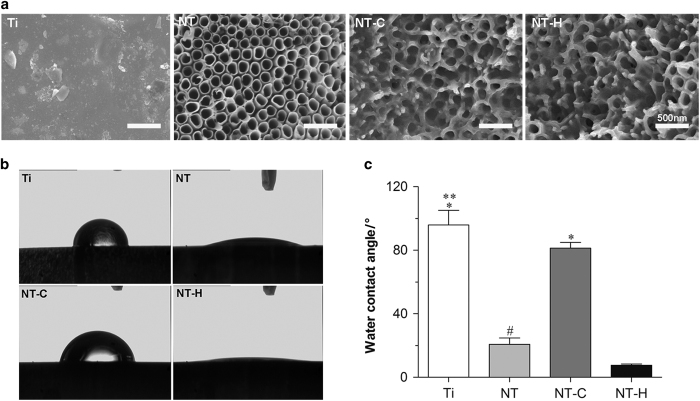
Surface characterization of the four different specimens. (**a**) Surface morphology using scanning electron microscopy (SEM). The surfaces of the drug-loaded nanotubes reserve the nanotubular structure. Magnification, ×50  000. The scale bar for the row is shown in the last image. (**b**), (**c**) Water contact angles of various specimens. *P<0.01, compared with titania nanotubes without drug-loading (NT) and HACC-loaded titania nanotubes (NT-H); **P<0.05, compared with chitosan-loaded titania nanotubes (NT-C); ^#^*P*<0.05, compared with NT-H.

The surface morphologies of Ti, NT, NT-C, and NT-H are shown in Figure 1. The diameter of the nanotubes was 160 nm, and the surfaces of the drug-loaded specimens retained the nanotubular structure as described above.

Figure 1 and Table 1 show the water contact angles of the four different specimens, which were 95.9±9.2°, 20.7±4.0°, 81.3±3.7°, and 7.6±0.8°, corresponding to Ti, NT, NT-C, and NT-H, respectively. These results indicate that the hydrophilicity of NT and NT-H was significantly improved compared with Ti and NT-C (*P*<0.01). Meanwhile, the hydrophilicity of Ti was also lower than NT-C (*P*<0.05). Table 1 shows the surface energy and adhesion work of these specimens. The surface energies of NT (65.6±5.8 J·m^−2^) and NT-H (72.4±1.1 J·m^−2^) were significantly higher than Ti (25.9±5.7 J·m^−2^) and NT-C (35.5±2.5 J·m^−2^) (*P*<0.01), and the adhesion work of NT (133.3±7.2 ΔE) and NT-H (144.9±1.2 ΔE) was significantly higher than Ti (66.8±11.8 ΔE) and NT-C (84.5±5.8 ΔE) (*P*<0.01), which is consistent with the results of the hydrophilicity evaluation.

### *In vitro* cytocompatibility evaluation

**Figure 2:**
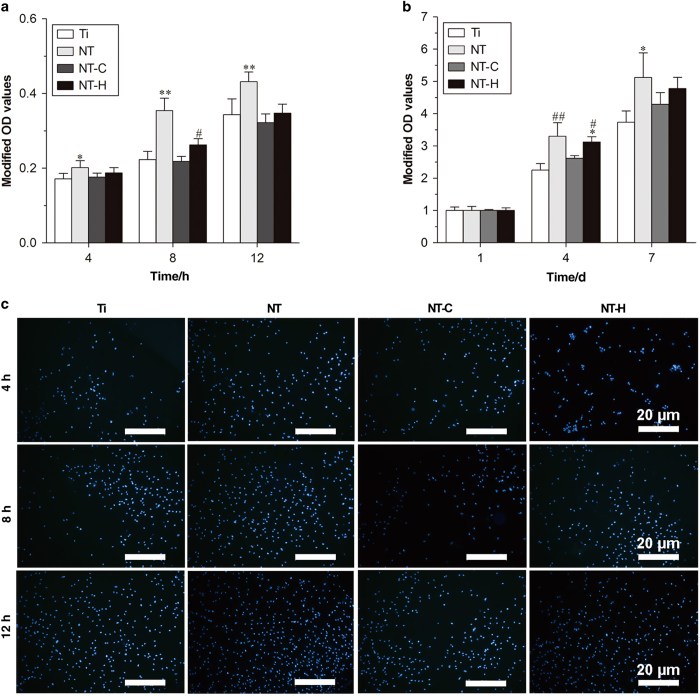
Attachment and proliferation assay of the human marrow-derived mesenchymal stem cells (hMSCs) on the four different surfaces. (**a**) Cell attachment on the samples assessed by the cell counting kit-8 assay. (**b**) Cell proliferation on various specimens. (**c**) Cell attachment on titanium without modification (Ti), titania nanotubes without drug-loading (NT), chitosan-loaded titania nanotubes (NT-C) and HACC-loaded titania nanotubes (NT-H) assessed by 4,6-diamidino-2-phenylindole staining after 4 8, and 12 h of culture. Magnification, ×100. The scale bar for the row is shown in the last image. *P<0.05, compared with Ti; **P<0.01, compared with the other groups; ^#^*P*<0.05, compared with NT-C; ^##^*P*<0.01, compared with Ti and NT-C.

**Figure 3:**
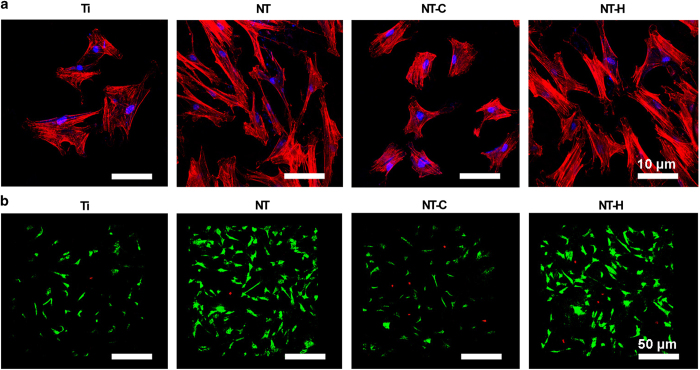
Cell spreading and viability on various specimens. (**a**) Cell morphology on the samples at 24 h, as observed by confocal laser scanning microscope (CLSM). Cells were stained with rhodamine phalloidin for the actin filaments (red) and 4,6-diamidino-2-phenylindole for the nucleus (blue). Magnification, ×400. (**b**) Cell viability evaluated by the Live/Dead assay after a 24-h incubation. Live cells with esterase activity appeared green, whereas dead cells with compromised plasma membranes appeared red. Magnification, ×100. The scale bar for the row is shown in the last image.

The results of the *in vitro* cytocompatibility evaluation are shown in Figures 2 and 3. As shown in Figure 2a, the counts of adhered cells on the surfaces of the NT was significantly higher than on the Ti at 4 h (*P*<0.05), and it could be observed that the cells adhesion on NT was better than the other groups at 8 and 12 h (*P*<0.01). Meanwhile, a lower counts of adhered cells was found on NT-C than on NT-H at 8 h (*P*<0.05). The cell proliferation measured by a CCK-8 kit on different samples is shown in Figure 2b and indicated that the cells on NT exhibited a higher relative proliferation rate than those on Ti and NT-C at 4 days (*P*<0.01). The relative proliferation rate of cells on NT-H was also higher than that on Ti (*P*<0.01) and NT-C (*P*<0.05) at 4 days. Moreover, the cell proliferation rate on Ti was lower than that on NT at 7 days (*P*<0.05) and no significant difference was found among NT and NT-H at the three time points (*P*>0.05). The numbers of cells stained with 4,6-diamidino-2-phenylindole on the surfaces of various specimens after 4, 8, and 12 h incubation, which is consistent with the results described previously, is shown in Figure 2c.

The cytoskeletons of the human mesenchymal stem cells on the surface of various specimens at 24-h incubation are shown in Figure 3a. Cells grown on NT and NT-H displayed polygonal, multilayer, clustering, and confluent morphology with more actin filaments linking adjacent cells, whereas the cells on Ti and NT-C had spindle, spherical, monolayer, and dispersive morphology with fewer actin filaments and poor spreading. The results indicated that cells on NT and NT-H showed well spreading compared with Ti and NT-C. In addition, a comparison of the cell viability of various specimens was performed by the Live/Dead Cell assay. As demonstrated in Figure 3b, the human mesenchymal stem cells displayed good cell viability in all four groups, with only a small number of dead cells detected in the NT-C and NT-H, which may be related to the slight cytotoxicity of the chitosan and HACC. Meanwhile, the cell confluence on the NT and NT-H was significantly better than the other two groups, which was consistent with the results analyzed above.

### *In vivo* anti-infection potential

#### Clinical manifestations

There were no deaths or severe systemic complications during the 42-day follow-up period after implantation. Five rats in the Ti group and four rats in the NT-C group showed clear local clinical signs of infection around the surgical sites post-surgery; however, the animals in the NT-H group exhibited no evident exudation or suppuration. The body weights of all animals increased gradually, and the body temperatures of all animals remained consistent and normal. There were no significant changes in the body weights and temperatures among the three groups (*P*>0.05).

#### Radiographical evaluation

**Figure 4:**
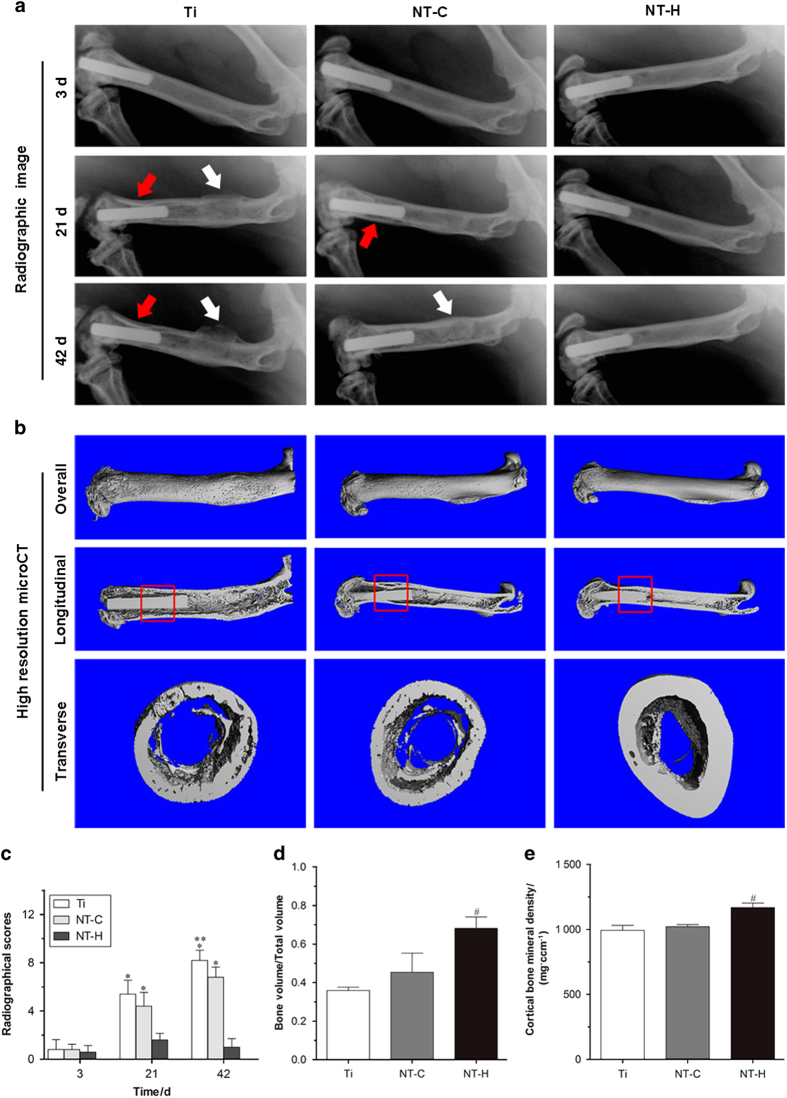
Radiographical images and evaluation. (**a**) Lateral X-rays of the left femur obtained at 3, 21, and 42 days during the follow-up period. Red arrows mark osteolysis, and white arrows indicate obvious periosteal reaction and new bone formation. (**b**) 3D micro-CT images of the left femur obtained from the overall, longitudinal, and transverse viewpoints at the time of sacrifice. The micro-CT evaluation of the middle femurs is confined to the red rectangle region. (**c**) Radiographic scores of the X-ray images. *P<0.01, compared with the HACC-loaded titania nanotubes (NT-H) (*n*=5); **P<0.05, compared with the titania nanotubes without drug-loading (NT) (*n*=5). (**d**) Bone volume/Total volume and (**e**) cortical bone mineral density of the selected regions of the left femurs evaluated by micro-CT. ^#^*P*<0.01, compared with the titanium without modification (Ti) and chitosan-loaded titania nanotubes (NT-C) (*n*=5).

The radiographic signs of obvious osteolysis, periosteal reaction, and articular surface destruction around the distal femurs in all animals of the Ti and NT-C groups were observed using X-ray 21 days post-implantation. The X-ray images obtained from the animals with NT-H rods did not exhibit obvious signs of articular surface destruction, osteolysis, or periosteal reaction (Figure 4a). Micro-CT analysis was used to confirm the radiographic appearance on the day of sacrifice (Figure 4b), and showed obvious implant loosening and porous changes in the femoral cortical bone in the Ti and NT-C groups, whereas better bone-implant contact and cortical integrity were observed in the NT-H group.

The quantitative analysis of the X-ray images in Figure 4c showed that the radiographic scores increased gradually after surgery for the Ti and NT-C groups, whereas the scores slightly increased at 21 days and then gradually decreased at 42 days in the NT-H group. The Ti and NT-C groups had significantly higher mean scores than the NT-H group (*P*<0.01) at 21 and 42 days after surgery, and the NT-C group exhibited lower mean scores than the Ti group at the time of sacrifice (*P*<0.05). In addition, the bone volume/total volume(Figure 4d) and bone mineral density (Figure 4e) of the NT-H group were significantly higher than those of the other two groups (*P*<0.01).

#### Microbiological assessment

**Figure 5:**
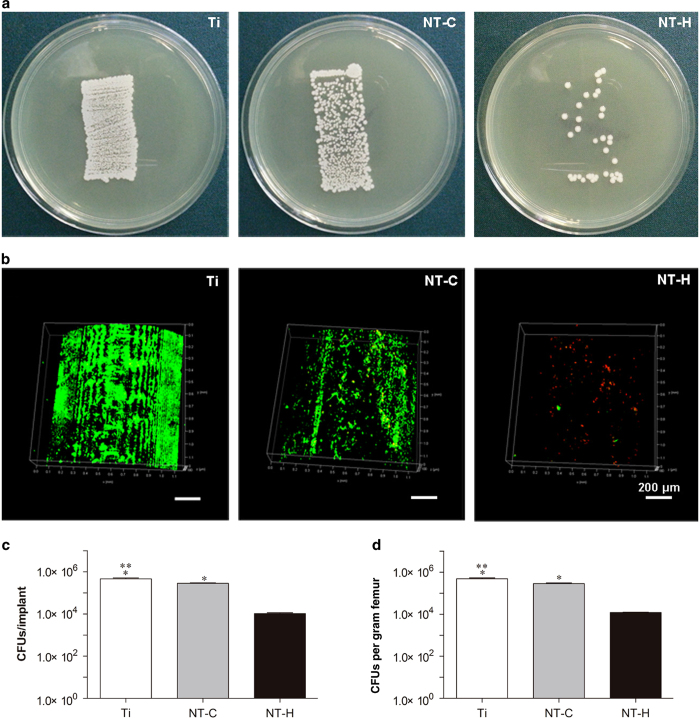
Microbiological evaluation of the implants and bone. (**a**) Roll-over cultures obtained from explanted rods. (**b**) Confocal laser scanning microscopy (CLSM) observation of explanted rods. Live bacteria showing green fluorescence were stained with SYTO 9 and dead bacteria showing red fluorescence were stained with propidium iodide. Magnification, ×100. The scale bar for the row is shown in the last image. (**c**) Amount of the detached adhered bacteria and biofilm after the rods were rolled over trypticase soy agar and (**d**) quantity of colony-forming units (CFUs) per gram of pulverized femur. *P<0.01, compared with the HACC-loaded titania nanotubes (NT-H) (*n*=6); **P<0.01, compared with chitosan-loaded titania nanotubes (NT-C) (*n*=6).

The rollover cultures of bacteria colonies that detached from the rods are shown in Figure 5a, indicating the lowest bacterial burden found in the NT-H rods among the three tested implants. In addition, the biofilm formation on the surface of different rods was observed directly by CLSM (Figure 5b). An obviously dense green fluorescence, indicating biofilm formation, was observed on the surfaces of the Ti and NT-C rods, with denser green fluorescence observed on the Ti rods. Conversely, considerably less green fluorescence and more discontinuous scattered red fluorescence was observed on the NT-H rods.

After sonication of the rolled implants, the results of the plate spreading demonstrated that the cultures obtained from the NT-H rods (1.07×10^4^±1.60×10^3^ CFUs per implant) had the lowest bacterial burden compared with the Ti (4.7×10^5^±8.0×10^4^ CFUs per implant) and NT-C (2.83×10^5^±3.06×10^4^ CFUs per implant) (*P*<0.01), and the mean colony counts for the NT-C rods were also obviously lower than those for the Ti (*P*<0.01) (Figure 5c). The bacterial burden per gram of femur also followed the trend of Ti group >NT-C group >NT-H group (Figure 5d), and the lowest bacterial burden was obtained in the NT-H group (1.22×10^4^±9.88×10^2^ CFUs·g^−1^) compared with the Ti (4.91×10^5^±9.89×10^4^ CFUs·g^−1^) and NT-C (2.9×10^5^±4.7×10^3^ CFUs·g^−1^) groups (*P*<0.01). The NT-C group also exhibited significantly lower CFUs/femur than did the Ti group (*P*<0.01).

**Figure 6:**
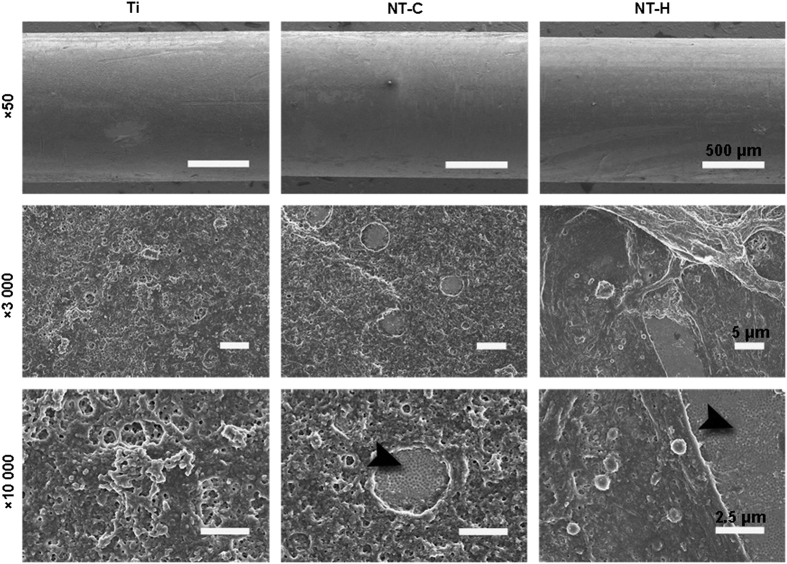
Scanning electron microscopy (SEM) observation of explanted implants. The black arrowheads indicate the intact nanotubular structure on the titanium rods. The scale bar for the row is shown in the last image.

Moreover, the SEM observation of the explanted rods further confirmed the results mentioned above (Figure 6); few scattered colonies and good spreading of osteoblasts were found on the surfaces of the NT-H rods. However, dense biofilm formation was found on the surfaces of the rods in the Ti and NT-C groups, especially in the Ti group, and the biofilm formation on the surfaces of the NT-C rods was separated discontinuously due to the nanofeatured surface topographies. The nanotubular structure of the titanium rods in the NT-C and NT-H groups remained unbroken after implantation, which confirmed the stability and feasibility of the drug-loaded nanotube structure *in vivo*.

#### Histopathological evaluation

**Figure 7:**
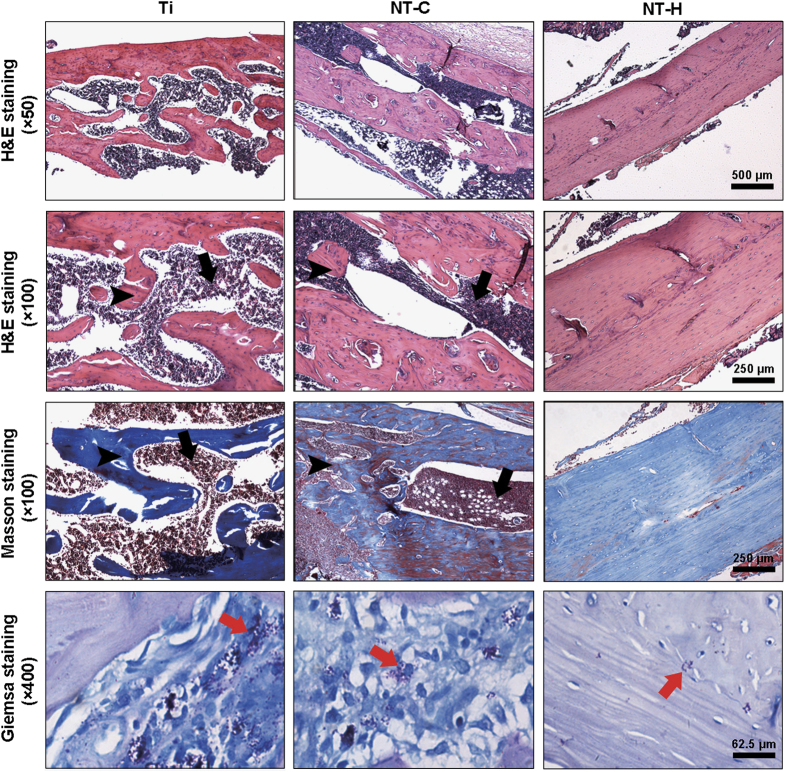
Representative histological images obtained from decalcified longitudinal sections without implants at the middle of the femur. Hematoxylin and eosin staining, and Masson’s trichrome staining were used to assess the changes in bone morphology, and Giemsa staining was used to determine bacterial contamination. Black arrows—intracortical abscesses or inflammatory cells; black arrowheads—massive enlargement and destruction of bone cortex; red arrows—bacteria. The scale bar for the row is shown in the last image.

The morphological changes in the longitudinal decalcified slices are assessed by hematoxylin and eosin and Masson’s trichrome staining, and the bacterial residue was confirmed by Giemsa staining. As presented in Figure 7, obvious signs of massive destruction of cortical bone, accompanied by intracortical abscesses and inflammatory cell infiltration, medullary sequestrum formation and fibrosis were found in the Ti and NT-C groups, and lots of bacteria, especially in the Ti group, were observed in the intramedullary cavities, as shown in the Giemsa slices at high magnification. In contrast, no evident abscess formation and significantly lightened bone destruction were observed in the NT-H group, and the number of bacteria colonized in the bone tissues decreased dramatically.

**Figure 8:**
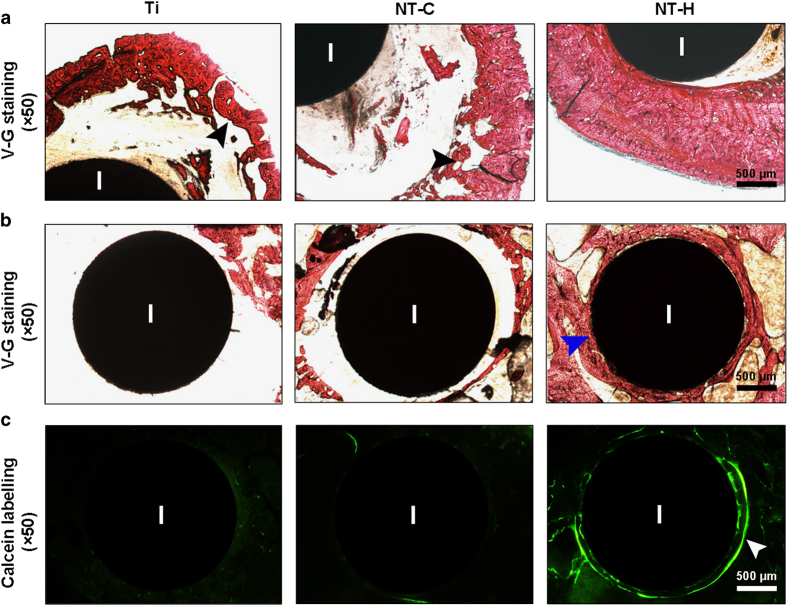
Representative histological images obtained from undecalcified transverse sections containing implants. Van Gieson staining was used to evaluate the (**a**) morphological changes in the cortical bone at the middle of the femur, and (**b**) the osteointegration around the implants at the condyle of the femur. (**c**) Fluorescent micrographs demonstrating new bone formation around the implants at 42 days after implantation. Black arrowheads, massive enlargement and destruction of the bone cortex; blue arrowheads, osteointegration around the implants; white arrowheads, obvious fluorescent deposition indicating new bone formation around the implants. I, implant. The scale bar for the row is shown in the last image.

The morphological changes in the cortical bone and osteointegration around the implants on the transverse undecalcified sections stained with Van Gieson are shown in Figure 8. There was obvious destruction of cortical bone in the Ti and NT-C groups, especially the expanded and thinner cortical bone in the Ti group. In contrast, the implants in the NT-H group were directly in contact with the surrounding bone with no obvious signs of cortical bone destruction, which is in accordance with the histopathological changes in the decalcified sections analyzed above. In addition, there was obviously better osteointegration and new bone formation around the NT-H implants at the femoral condyle than in the other two groups, which further confirmed the *in vivo* cytocompatibility of the HACC-loaded TNTs.

## Discussion

The development of implant-associated infection is one of the most devastating complications in fracture fixation surgery, and it sometimes results in poor osteointegration, chronic osteomyelitis, and finally implant failure. Furthermore, implant removal and multiple debridements are often necessary, which results in an unacceptable economic and psychological burden for both physician and patient.^32^

Implant surfaces favor bacterial adhesion, colonization, and biofilm formation after implantation, and the dose of contaminating colonies required to cause infection is low when the surgical site contains foreign material, according to several reported experimental models.^33,34^ Furthermore, there is an immuno-incompetent fibro-inflammatory zone in the interstitial milieu around the implants, which is susceptible to bacterial colonization and favorable to the recurrence of infection.^35,36^ After the initial adhesion of bacteria to implant surfaces during a post-implantation period of 4–6 h, cell aggregation and accumulation occur to form a mature biofilm, which exhibits enhanced antibiotics resistance and increased protection from the host defense.^15,37^ Therefore, bacterial adhesion inhibition is of great importance to the manufacture of anti-infection implants.

The surface characteristics of biomaterials have been supposed to be important to bacteria and cell adhesion during the “race to the surface” competition. Hydrophobicity plays a critical role in a wide range of bacterial infection.^38^ Hydrophobic surfaces exhibit a trend of bacterial adhesion due to nonspecific adsorption of proteins and hydrophobic-hydrophobic interactions with bacterial surfaces, and it is supposed that reduced interaction between bacteria and a hydrophobic matrix could be achieved by increasing surface hydrophilicity.^39–41^ Our results revealed that the hydrophilicity of NT and NT-H was significantly improved compared with Ti, which indicates that the nanotube structure formed on the titanium substrate increased the hydrophilicity of the surfaces. Meanwhile, the relatively high hydrophobicity observed on the NT-C surfaces may be caused by the poor water solubility of chitosan. Accordingly, the surface energy and adhesion work of NT and NT-H are also significantly higher than Ti and NT-C, which is believed to be meaningful to bacterial adhesion inhibition, as previously reported.^42^ Meanwhile, our *in vitro* cytocompatibility assessments revealed that NT and NT-H displayed good osteogenic activity when compared with Ti and NT-C, which indicates that the vitality of human mesenchymal stem cells is not obviously affected by the loaded HACC and high hydrophilicity may be beneficial to the attachment of osteogenic cells.

However, there are opposite conclusions in regard to the relationship between the surface chemistry of the biomaterial and its antibacterial properties, which showed a positive correlation between the hydrophilicity and bacterial adherence.^43,44^ Therefore, it is important to evaluate the *in vivo* antibacterial properties of the nanotube structure and the drug-loaded TNTs. Our *in vivo* results demonstrated that the chitosan-loaded TNTs showed obvious development of bone infection, which indicates that both the nanotube structure and surface characteristics exhibited limited *in vivo* antibacterial activity over the course of 6 weeks post-surgery. The NT-H group showed good implant-bone contact, cortical integrity and dramatically decreased bacterial load; however, evident bone destruction and bacterial burden were observed in the Ti and NT-C groups. Although the *in vitro* antibacterial activity of the chitosan-immobilized titanium surface was investigated and verified by a previous report,^45^ our present study indicated that it was still insufficient to prevent the development of bone destruction due to the aggravated implant-associated infection *in vivo* when compared with NT-H. Various modifications were utilized to improve the antibacterial activity of chitosan.^46,47^ Our previous work also found that the nanotube arrays generated on the implants exhibited alleviated bone infection *in vivo*, which was similar to the antimicrobial performance of the NT-C in this study.^14^ Therefore, it was necessary to use the HACC-loaded nanotube structure to prevent the development of bone infection *in vivo.* Although the hydrophilic interfaces of the biomaterials played positive roles in the bacterial adherence and biofilm formation inhibition in this study, the *in vivo* anti-infection potential of the implants depends on the antibacterial agents loaded in the nanotubes. In addition, the bacteria inoculation (1×10^5^CFUs) in the medullary cavity of the distal femur in this animal model was much higher than the clinical amount of contaminating bacteria in the surgical site of the implantation,^23^ which may be an important cause of the residual bacteria detected in the NT-H group. According to our previous *in vitro* research, the total amounts of HACC released from nanotubes with a diameter of 160 nm over 60 h was 1 080 μg,^24^ which was above the minimal inhibitory concentration of the tested strain (64 μg·mL^−1^). Thus, additional systemic antibiotic administration may be necessary to completely eradicate the bacteria, and modification of diameter of nanotubes, which can regulate the drug-loading, is a feasible and effective method to optimize the *in vivo* anti-infection properties of HACC-loaded TNTs. The nanotubular structure of the NT-C and NT-H groups remained intact after the implantation, as displayed at high magnification, which indicated *in vivo* stability of the nanotubes, and therefore provides reliable evidence for the viability of drug-loaded nanotube implants in orthopedic surgery.

## Conclusions

In summary, our study demonstrated that HACC-loaded nanotubes on titanium implants exhibited obvious anti-infection potential *in vivo* and good osteoactivity *in vitro* when compared with Ti and NT-C. The nanotube structure and surface characteristics exhibited limited antimicrobial effects on the implant-associated infections *in vivo*.

## Figures and Tables

**Table 1:** Contact angles, surface energy, and adhesion work of the four different samples

Specimen	Contact angle/°	Surface energy/(J·m^−2^)	Adhesion work/aΔE
Ti	95.94±9.17	25.87±5.68	66.48±11.81
NT	20.74±4.05	65.59±5.75	133.32±7.24
NT-C	81.34±3.65	35.48±2.47	84.52±5.79
NT-H	7.60±0.80	72.43±1.12	144.89±1.18

NT, nanotubes without polymer loading; NT-C, nanotubes loaded with chitosan; NT-H, HACC-loaded nanotubes; Ti, titanium implant.

a ΔE=*γ*_*lv*_+*γ*_*sv*_−*γ*_*sl*_. Where *γ*_*lv*_indicates the surface tension of the liquid, *γ*_*sv*_indicates the surface tension of the solid, and *γ*_*sl*_represents the solid-liquid interfacial tension.
